# A Few Clinical Notes on the Usual Situation of Spinal Hemorrhage, Which Results from Traumatic Influence, with Report of Four Typical Cases

**Published:** 1893-10

**Authors:** Thomas H. Manley

**Affiliations:** New York


ATLANTA
MEDICAL AND SURGICAL JOURNAL.

VOL. X. OCTOBER, 1893. NO. 8.

EDITED BY
LUTHER B. GRANDY, M. D., AND MILLER B. HUTCHINS, M. D.,
WITH THE CO-OPERATION OF
H. V. M. MILLER, M. D., LL. D., VIRGIL O. HARDON, M. D.,
FLOYD W. McRAE, M. D., AND ARTHUR G. HOBBS, M. D.

### *ORIGINAL COMMUNICATIONS.*

### A FEW CLINICAL NOTES ON THE USUAL SITUATION OF SPINAL HEMORRHAGE, WHICH RESULTS FROM TRAUMATIC INFLUENCE, WITH REPORT OF FOUR TYPICAL CASES.

BY THOMAS H. MANLEY, M. D.,
NEW YORK.

With a personal experience in more than seventy cases of serious spinal injuries; with fifteen autopsies, in eleven of which I have full notes; most of which cases treated in hospital, I am in a position to support by theoretical reasoning with practical demonstration; particularly in connection with the question of the usual and more common situation of hemorrhagic leakage in traumatisms, which involve the anatomical structures of the spinal canal or cord.

In the main, these observations have led me to the conclusion that in no case of injury to the back, resulting from physical violence, are we liable to declare with certainty, immediately after the application of force, that the spinal cord or its serous envelopes have sustained serious damage, unless some phase of paralysis distinctly supervene.

When the phenomenon immediately, or within twenty-four hours after injury follows, it invariably indicates either tension, pressure or disorganization of the meninges, or the medullary substance of the cord.

As the element of pressure, alone, in the vast majority of cases preponderates, a few comments on it only, with the report of four cases, will be considered at present.

Medullary pressure may be exclusively vertebral, as in fracture or dislocation. It will be wholly intervertebral when the lesion is limited to that network of plexuses which ramify through, and arch across, the loose cellular tissues, in the osseo-thecal space, from the atlas to the sacro-coccygeal articulation.

I have never met with a single case, on autopsy, which gave conclusive demonstration of a free hemorrhage, which was exclusively limited to the spinal marrow, or sub-dural space, in which the leakage was of sufficient volume to seriously threaten the integrity of the cord by immediate pressure, or to excite consecutive inflammation. On the contrary, in all cases of spinal hemorrhage, in which *post-mortem* examinations were permitted, the blood escape was external to the theca; extra-dural.

We can seldom have extra-vertebral hemorrhagic pressure as an independent factor in spinal injuries. When such does exist, it is always in association with either fracture or dislocation.

Hemorrhage into the spinal canal—extra-dural—as a source of meningeal inflammation, or medullary compression, in traumatisms of every description in point of frequency, vastly preponderates.

The physical quality of this pressure is hydrostatic; anatomically consisting in most cases of arterial blood in a fluid or coagulable state.

The ultimate fate of one, the subject of traumatic spinal-hemorrhage, will depend on various factors, the most important of which will have reference:

1. With respect to situation and extent.

2. The suddenness of its onset, or its insidious development.

3. The general condition and complications.

The most favorable termination in any given case will be by prompt resorption, which is the rule when the leakage is limited in quantity. When this fails a compression meningitis, or meningo-myelitis, is certain to follow, with a succeeding paralysis of varying duration, according to the extent of hemorrhage and the segment of the cord involved. This has an invariably mortal termination.

These latter sequelæ of extra-dural hemorrhage, by a rapidly ascending spread of inflammatory changes, soon involve the respiratory reflexes, ending life by a neural inhibition.

With the preceding preparatory notes on a few of the grosser lesions peculiar to one phase, chiefly of intravertebral pressure, I herewith subjoin in epitome the histories of four cases of very serious injuries which came under my care during the past summer ('92) at the Harlem Hospital.

CASE 1.—P. G., admitted to hospital July 4th, age forty-three years.

*Diagnosis.*—Hemorrhage into the lower cervical, upper and dorsal regions of the spinal canal.

*History.*—On the night of July 3, patient retired at about 9 o'clock. An hour later, while in a somnambulistic state, patient arose from bed, and, while groping about his room fell from the window to the ground, a distance of about twelve feet.

He was found later, where he fell, in an unconscious state. The following day he was sent to the hospital.

At this time he had regained consciousness. But he had complete paraplegia with incontinence of feces and urine. Great pain in the back. His urine was strongly ammoniacal, and that withdrawn was tinged with blood.

His temperature was 100⅓° and pulse 102 ; weak.

In spite of treatment, which was but palliative, he went on from bad to worse, until July 13, when temperature went up to 103½°. Central symptoms were now well marked, and as the respiratory muscles became involved, he gradually became weaker and sank early that evening.

*Autopsy.*—Twenty-four hours after death contusion of the spinal-column, our diagnosis, was confirmed. The spinal-canal, external to the meninges, stuffed from end to end by a large sanguinous effusion.

On division of the dura-mater, its inner surface was seen coated by a thin sanguino-serous exudate, nearly throughout its entire extent. The medullary substance of the cord in places was found compressed and softened. The pia-mater was on its anterior surface dotted by particles of pus; and in places was thickened and congested. Cranium not opened.

CASE 2.—W. D., age forty-three years; admitted to hospital July 25.

*Diagnosis.*—Fracture of spinous apophysis of ninth dorsal vertebra with spinal hemorrhage.

*History.*—Patient, while at work in a lumber yard, was violently struck in the back by the end of a heavy falling piece of timber, which came from a considerable height. He was felled by the blow, and, on attempting to rise, discovered that he was powerless in the lower limbs. He immediately commenced to vomit freely, and manifested other well pronounced symptoms of severe shock. He was admitted to hospital the same day, when he had complete motor paralysis of the lower extremities, though sensation was not altogether abolished. He had marked gastric disturbance, with very severe pain in the back and incontinence of the bladder and rectum. For the pain morphine had to be given in large and repeated doses.

July 26. In great pain, but general condition improved. Now able to move his feet.

On the afternoon of this day, patient was etherized, when, without difficulty, both posterior lateral arches of the fifth dorsal vertebra, which were found fractured, were lifted out. Although these fragments were completely broken off at the transverse processes, there was no displacement of them, or pressure on the cord. With the scissors and forceps they were easily detached and lifted out.

When these broad osseous plaques of bone were removed, the bare, white, unbroken surface of the dura-mater came into view, so that it could be distinctly seen, and easily felt with the finger, through the hiatus occasioned by their removal.

The dura-mater showed no traces of injury.

Now the wound was partially closed, drained and buried under a mass of fluffy dressings, when the patient was returned to bed.

July 27. Although his epileptic symptoms had, on this date, presented evidence of improvement, his general condition was much worse. At 12 M. temperature 103¾°, pulse 122.

July 28. Temperature at 8 A. M., 104¼°, pulse 150.

Died two hours later; persistent and ungovernable vomiting continuing with unabated severity to the last. Death was by a general exhaustion and sudden breaking up of the vital powers rather than to any condition dependent on paralysis.

*Autopsy.*—Twenty-six hours after death, the spinal canal was opened by myself, when it was found that there had been a large hemorrhage—extra-dural—which had been caused by a laceration of the meningo-rhachidian vessels on both sides of the cord, at the seat of fracture. The blood was firmly coagulated and filled the greater part of the canal, above and below the seat of fracture. There was no hemorrhage into the medullary substance. The evidence of incipient meningeal inflammation was seen, as in the preceding case, though not so well accentuated.

CASE 3.—J. W. J., age thirty-five years, August 1, 1892.

*Diagnosis.*—Hemorrhage into spinal cord, lower cervical region.

*History.*—On August 1, while under the influence of liquor, on descending stairs, lost his footing and fell heavily on his neck and shoulders.

On admission to hospital it was found that he had complete paralysis from the cervico-dorsal junction downward, with very little power in the arms, the right particularly. As in the preceding cases, motor paralysis predominated from the beginning. For eight days there was littie change in his condition, although at no time did the temperature mount beyond 100, or his pulse above 92.

August 9. Commenced to show signs of recovery from paralysis, with improvement in his general condition.

By the use of Sagre's gallow and Meig's case's spinal-carriage, other general measures and good diet, he so much recovered himself as to be able, after a time, to walk unassisted, dress and undress himself. He left the hospital for home September 28. He has since perfectly recovered.

CASE 4.—E. W., age twenty-five years. American.

*Diagnosis.*—Hemorrhage into spinal canal.

*History.*—Admitted on September 8, 1892. On September 6 patient was thrown from a vendor's wagon, while his horse, which was running away, was turning the corner of a street.

On examination, it was found that he had complete motor-paralysis of the lower extremities, with marked diminution in sensation. He was able to use his arms only to a slight extent.

September 8. Total loss of sensation, as far up as the second rib. The power of motion now was entirely lost in the upper and lower extremities. Temperature 99.2°, pulse 52.

September 9. Patient's general condition much more serious.

September 10. The morning temperature suddenly rose to 102½, and symptoms of rapidly developing pulmonary-edema were present.

September 13. Becoming steadily worse, and died on evening of this date.

*Autopsy.*—Twenty-four hours after death spinal canal was opened and a large extra-dural hemorrhage was discovered occupying the greater part of the entire canal. Such extensive pathological changes were seen in the meninges and substance of the cord as indisputably demonstrated that inflammatory changes had become well established. We were not permitted to examine the brain in this or in the two preceding autopsies.

COMMENTS.

In the above short narrative but a very incomplete report of the varied clinical manifestations and pathological changes encountered in intravertebral hemorrhage has purposely been submitted, as on a later occasion I intend to fully and methodically exhaust the subject; for, in the present instance, my aim has been to offer but a few elementary outlines.

Nevertheless, while on this subject, I may say that this whole question of spinal-hemorrhage of a traumatic origin, is a veritable *terra incognita;* for it is apparent, from the most superficial examination of most of the average text-books on general surgery, that almost nothing can be found on intravertebral hemorrhage. In works on surgical pathology, too, and even those exclusively devoted to neurology, there is a dearth of information on sanguinous effusions of the description under consideration.

At a late meeting of the Neurological Section of the Academy, the position was taken that hemorrhage into the parenchyma of the cord was a most prolific source of acute myelitis in pathological conditions; and it was alleged that this condition, in many instances, was attributable to injury.

For the first time in my life I learned that the medullary substance of the cord was permeated by more than two hundred arteries. Now, I most emphatically repudiate the allegation that the medullary substance of the cord can, in any sense, be regarded as a highly vascular structure, as nearly its sole blood supply is from above, through very small recurrent branches of the vertebral arteries which split up as they descend, and are as freely distributed at the commissures and cornua in the meshes of the pia-mater, as in the white and gray substance of the cord.

Reasoning from analogy and from knowledge of the various vascular diseases incident to certain constitutional derangements or degenerative changes, it is easy to understand how we may in certain pathological conditions of the cord have a hemorrhage of a very limited extent from the venous-radicles, the arterioles or capillaries. But that we can ever have a primary hemorrhage of any tangible proportion, as a consequence of physical violence, into the medullary substance without extensive destruction of the overlying soft parts and the bony canal, has not yet been demonstrated.

In six hundred and forty-two cases of serious spinal injuries recorded in the surgical history of the war of the late rebellion, not a single instance is cited of a primary, uncomplicated hemorrhage into the medullary substance though there is a considerable number recorded of extra-thecal extravasation of blood within the vertebral walls.

The precise knowledge of the extent and situation of rhachidian hemorrhage is a matter of considerable importance, both from a forensic and therapeutical standpoint.

Although the study of the lesions consecutive to localized intra-vertebral hemorrhagic pressure is both an attractive and fruitful one, the limit of this contribution permits me only to make a most cursory and superficial survey of the subject.

